# Temperature Dependence of Stress and Optical Properties in AlN Films Grown by MOCVD

**DOI:** 10.3390/nano11030698

**Published:** 2021-03-10

**Authors:** Wenwang Wei, Yi Peng, Jiabin Wang, Muhammad Farooq Saleem, Wen Wang, Lei Li, Yukun Wang, Wenhong Sun

**Affiliations:** 1 Research Center for Optoelectronic Materials and Devices, School of Physical Science & Technology, College of Chemistry and Chemical Engineering, Guangxi University, Nanning 530004, China; 1814404039@st.gxu.edu.cn (W.W.); 1907401031@st.gxu.edu.cn (Y.P.); 1907401030@st.gxu.edu.cn (J.W.); farooq@tju.edu.cn (M.F.S.); 1807301028@st.gxu.edu.cn (L.L.); 20180116@gxu.edu.cn (Y.W.); 2 Advanced Micro-Fabrication Equipment Inc., Shanghai 201201, China; wenwang@amecnsh.com; 3 Guangxi Key Laboratory of Processing for Non-Ferrous Metallic and Featured Materials, Guangxi University, Nanning 530004, China

**Keywords:** AlN films, nano-patterning, stress, optical properties, Raman spectroscopy, HRXRD, TEM, spectroscopic ellipsometry

## Abstract

AlN epilayers were grown on a 2-inch [0001] conventional flat sapphire substrate (CSS) and a nano-patterned sapphire substrate (NPSS) by metalorganic chemical vapor deposition. In this work, the effect of the substrate template and temperature on stress and optical properties of AlN films has been studied by using Raman spectroscopy, X-ray diffraction (XRD), transmission electron microscopy (TEM), UV-visible spectrophotometer and spectroscopic ellipsometry (SE). The AlN on NPSS exhibits lower compressive stress and strain values. The biaxial stress decreases from 1.59 to 0.60 GPa for AlN on CSS and from 0.90 to 0.38 GPa for AlN on NPSS sample in the temperature range 80–300 K, which shows compressive stress. According to the TEM data, the stress varies from tensile on the interface to compressive on the surface. It can be deduced that the nano-holes provide more channels for stress relaxation. Nano-patterning leads to a lower degree of disorder and stress/strain relaxes by the formation of the nano-hole structure between the interface of AlN epilayers and the substrate. The low crystal disorder and defects in the AlN on NPSS is confirmed by the small Urbach energy values. The variation in bandgap (*E_g_*) and optical constants (*n, k*) with temperature are discussed in detail. Nano-patterning leads to poor light transmission due to light scattering, coupling, and trapping in nano-holes.

## 1. Introduction

AlN is a III-V semiconductor that is well-known for its direct wide bandgap (~6.2 eV), high thermal conductivity, high electrical resistivity, high breakdown voltage, and excellent piezoelectric and optical properties. Its lattice constant and the thermal expansion coefficient values are very close to other III-nitride materials that determine its applications such as GaN epitaxy, ultraviolet light sources, radiation detectors, microwave devices, photoelectric devices, power electronic devices and surface acoustic wave devices [1,2,3,4,5,6,7]. Compared with sapphire or SiC substrates, AlN has higher chemical compatibility with AlGaN and lower lattice and thermal expansion mismatch [8,9,10]. Therefore, AlN epilayers are also commonly used as buffer layers to improve the structure of heteroepitaxial films in epitaxial growth. The use of an AlN buffer layer and an AlN/AlGaN superlattice is reported to improve the power level and device performance of LEDs due to the reduction of defects and cracks [9]. A thick AlN layer grown on a trench-patterned AlN/sapphire template results in a crack-free and smooth surface, which is desirable for LEDs [11]. Substantial improvement in the device lifetime and reliability can be achieved by fabricating deep ultraviolet (DUV) LED structures over fully coalesced thick AlN films [12]. High quality AlN ceramics have been achieved for the nuclear energy industry by irradiating AlN with heavy ions [13].

Sapphire is widely used as a substrate of choice for AlN epitaxial growth because of its easy availability and low cost. AlN films have been grown by various methods [14,15,16,17,18,19,20], such as chemical vapor deposition (CVD), pulsed sputter deposition (PSD), pulsed laser deposition (PLD), molecular beam epitaxy (MBE), reactive magnetron sputtering, hydride vapor phase epitaxy (HVPE), and metal organic chemical vapor deposition (MOCVD). CVD is found to be the most suitable technique in controlling the preferred orientation [21]. The AlN crystal planes oriented parallel to the substrates can change with increasing total gas pressure and decreasing deposition temperature due to supersaturation in the gas phase [14]. The PSD technique is useful for the growth of high-quality AlN films at room temperature, however, the AlN films obtained by this technique exhibit considerable residual compressive strain [15]. PLD as a deposition method, contains molten small particles or target fragments in the deposited films, which greatly reduces the quality of the films [22]. High quality AlN films can be obtained by molecular beam epitaxy, but it is not suitable for thick films due to its slow growth rate [23]. Although the HVPE method has an extremely high growth rate, the high defect density is a serious drawback of this technique [24]. Among above these methods, the growth rate of MOCVD method is moderate and the crystal quality is high. During the MOCVD growth, the high thermal stability of NH_3_ requires the use of high substrate temperatures, typically more than 900 °C for AIN, and the nitrogen loss can be partially alleviated by the use of high III/V gas phase ratios (for example, >2000:1) [25]. MOCVD is one of the most mature methods to grow AlN films due to its high growth rate, high quality yield, high production capacity and low cost.

In the crystal system of AlN (*a* = 0.3110 nm, *c* = 0.4980 nm, *α_a_* = 4.2 × 10^−6^ K^−1^) and sapphire (*a* = 0.4758 nm, *c* = 1.2291 nm, *α_a_* = 7.5 × 10^−6^ K^−1^), the lattice mismatch is up to 13.2% and thermal expansion coefficient mismatch is as high as 45.4% [6,8,26]. The large lattice and thermal mismatch lead to stress in the epitaxial layer. The residual stress is an important factor affecting the properties of AlN films. Moreover, better stress and optical properties of AlN films are crucial for device performance. The use of nano-patterned sapphire as a substrate can effectively reduce stress caused by lattice mismatch, and alleviate the dislocation density that can be reduced by 1–2 orders of magnitude [27,28,29]. In this paper, the stress and optical properties of AlN films on flat and nano-patterned sapphire substrates (NPSS) are compared and analyzed in order to improve the quality of AlN for applications in high-quality UV-LEDs.

## 2. Materials and Methods

**Figure 1:**
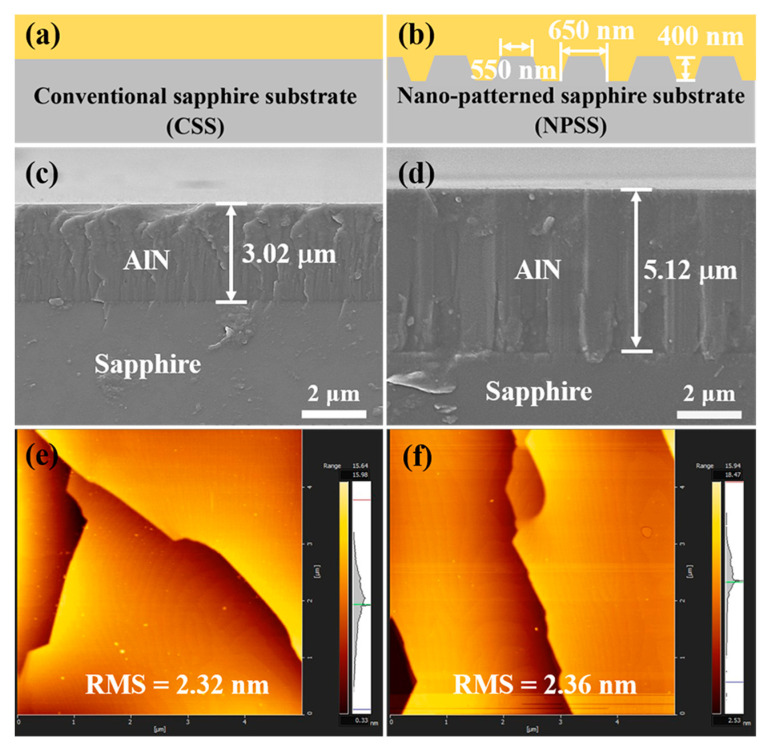
Schematic diagram of (**a**) conventional sapphire substrate and (**b**) nano-patterned sapphire substrate. Morphology scanning electron microscopy (SEM) cross-section images of AlN/sapphire films: (**c**) sample A and (**d**) sample B. Atomic force microscopy (AFM) surface micrographs are shown in (**e**) sample A and (**f**) sample B.

AlN samples were grown using the AMEC Prismo HiT3^TM^ MOCVD platform on a 2-inch [0001] conventional (flat) sapphire substrate (sample A) and a nano-patterned sapphire substrate (sample B). The details of growth information and thickness of AlN samples are given in Table 1. The schematic diagrams of samples A and B are shown in Figure 1a,b. The nano-patterned substrate has 400 nm deep truncated cone patterns with a 550 nm diameter at the top circumference and 650 nm at the bottom. The pitch length is ~1000 nm. The sapphire substrates were coated with ~15 nm AlN layer by physical vapor deposition (PVD). For the epitaxial growth in MOCVD, trimethyl-aluminium (TMAl) and ammonia (NH_3_) were used as Al and N precursors, respectively. H_2_ was the carrier gas. Firstly, the sapphire substrates with a PVD AlN nucleation layer were heated up to 1150 °C in H_2_ ambient, followed by the initial AlN roughing layer growth. The temperature was then ramped up to 1250 °C to recover the surface and maintain two-dimensional growth. Subsequently, the AlN samples were annealed for 25 min at 1550 °C with N_2_ as the ambient gas.

The morphology of the AlN surface was investigated by atomic force microscopy (AFM; 5100 N). The thickness of the AlN epilayer was determined by scanning electron microscopy (SEM; Hitachi SU8220) and additionally by spectroscopic ellipsometry (SE; ME-L) for further confirmation. The variations of bandgap (*E_g_*) and optical constants (*n, k*) with temperature were also estimated by SE measurements. In the spectral range of 195–1650 nm (0.75–6.35 eV), the measurement of variable angle spectroscopic ellipsometry (VASE) was carried out by using a dual rotating compensation Mueller matrix ellipsometer and the variable temperature was determined by an embedded thermocouple with temperature from 300 to 850 K. The analysis of VASE experiment data was used to obtain optical constants, bandgap, thickness and roughness of AlN films. The crystal quality and stress related properties of the epitaxial layers were characterized by high-resolution X-ray diffraction (HRXRD; X’Pert^3^ MRD) and Raman spectroscopy (LabRAM HR Evolution). Temperature dependent Raman measurements were performed by using a 532 nm of He/Cd laser excitation source with a back-scattering geometry. HRXRD data was obtained with Ge (220) four-crystal monochromator using a Cu K_α1_ = 1.5406 Å radiation with an angular resolution of about 12 arcsec for AlN films. Transmission electron microscopy (TEM; FEI Talos F200X) images were obtained by using a thermal field emission operating at 200 kV to examine the microstructural properties of the AlN epilayers grown on the different substrate templates. The dual-beam ultraviolet-visible spectrophotometer (UV-Vis; TU1901) was employed to determine optical transmission spectra in the wavelength region from 190 to 850 nm. The spectra were recorded with a resolution of λ = 0.01 nm and photometric accuracy of ±0.3%.

## 3. Results and Discussion

### 3.1. Morphology Analysis

The SEM images were used to determine the thicknesses of AlN epilayers. Figure 1c,d represents the SEM cross-sectional images of samples A and B; the corresponding thicknesses are ~3.02 and ~5.12 μm, respectively. AFM has proved to be a very useful and powerful technique for correctly determining the surface roughness, surface energy and morphology [30,31]. The surface morphology of samples A and B is observed by AFM in the 5 × 5 μm^2^ region, as shown in Figure 1e,f, and the values of root mean square (RMS) surface roughness of films are 2.32 and 2.36 nm, respectively. It can be seen from the figures that the surface of AlN is in the shape of steps.

### 3.2. Raman Spectroscopy Analysis

**Figure 2:**
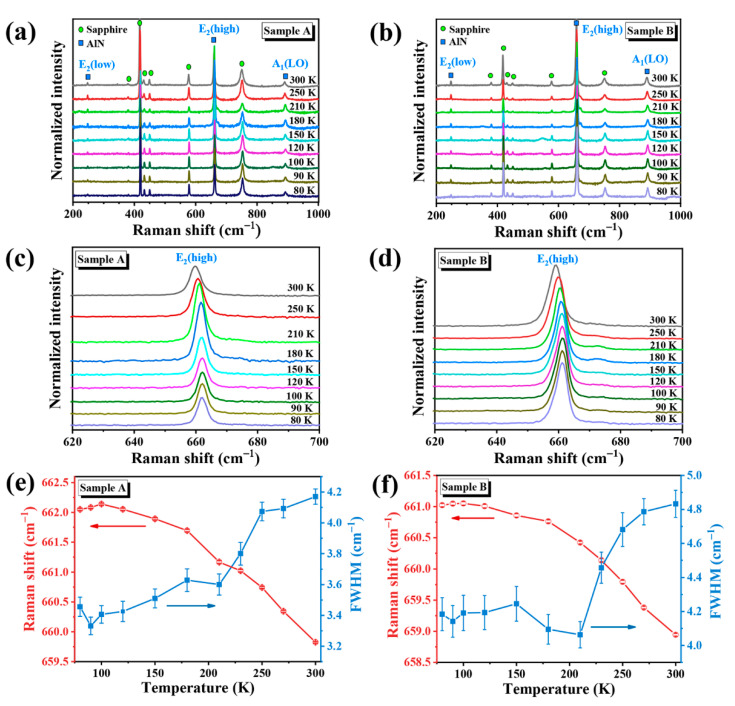
Raman spectra of AlN/sapphire films were taken at variable temperatures from 80 to 300 K by using 532 nm laser excitation, where (**a**,**b**) correspond to the sample A and sample B, respectively. The Raman shift of E_2_(high) phonon mode for (**c**) sample A and (**d**) sample B. Raman shift and full width at half maximum (FWHM) versus temperature of the E_2_(high) phonon mode for (**e**) sample A and (**f**) sample B in the range of 80−300 K.

The Raman spectra of AlN films were obtained in the temperature range 80–300 K as shown in Figure 2a,b. For sample A/B, the room temperature Raman spectra show peaks at 247.4/247.8, 659.8/658.9 and 890.0/889.6 cm^−1^ corresponding to the E_2_(low), E_2_(high), and A_1_(LO) phonon modes of wurtzite AlN, respectively. The Raman peaks at 379.3/379.5, 417.7/417.9, 430.2/431.0, 577.1/577.4 and 750.3/750.5 cm^−^^1^ are associated with the underlying sapphire substrate. It is known that in the back-scattering geometry, the E_2_ and the A_1_(LO) modes are allowed for AlN (0002), whereas the A_1_(TO) and E_1_(TO) phonon modes are forbidden. Here, a weak E_1_(TO) phonon mode (~671.3 cm^−^^1^) is also observed for both samples at low temperatures as shown Figure 2c,d; that is associated with the presence of slightly misaligned islands [32,33]. The E_2_(low) corresponds to the phonon mode when atoms are in shear motion. The peak of E_2_(high) is redshifted with the increase of temperature. The E_2_(high) mode typically is used to characterize the residual stress in AlN. The lattice mismatch and difference in the thermal expansion coefficients between the substrate and the AlN layer leads to stress in the epitaxial layer [17]. The residual stress affects the phonon scattering frequency. A blueshift in E_2_(high) phonon frequency is used as an indication of compressive stress, whereas a redshift corresponds to tensile stress [34].

The full width at half maximum (FWHM) and positions of the E_2_(high) peak derived by Lorentz fitting are plotted as a function of temperature (80–300 K), as shown in Figure 2e,f for samples A and B, respectively. The FWHM of the E_2_(high) peak of AlN is a useful indicator of crystallinity [35]. The FWHM of E_2_(high) peak for sample A increased from 3.33 to 4.17 cm^−^^1^, and that of sample B increased from 4.06 to 4.83 cm^−1^ in the temperature range of 80–300 K. As the temperature range is sufficiently low for defect formation or any variations in crystal quality to occur, the increase in FWHM with temperatures can be assigned to mismatch and alignment disorder.

As can be seen in Figure 2e, for the E_2_(high) peak of sample A, the Raman shifts are larger than that of strain-free AlN (ω_0_ = 657.4 cm^−^^1^), indicating the compressive stress induced by substrate. The larger blueshift at low temperature indicates that the compressive stress is larger at low temperature. It can be explained in terms of thermal expansion of the lattice and the anharmonic effect of phonon–phonon interactions that result in the redshift with temperature [36,37]. Thus, heating effectively promotes strain relaxation. The relatively smaller frequency shifts for sample B indicate lower values of stress due to the nano-patterning of the substrate. The nano-holes provide a channel for the gradual release of the stress to a nearly stress-free state accompanied by the lateral growth process of the AlN columns on the mesas [38].

**Figure 3:**
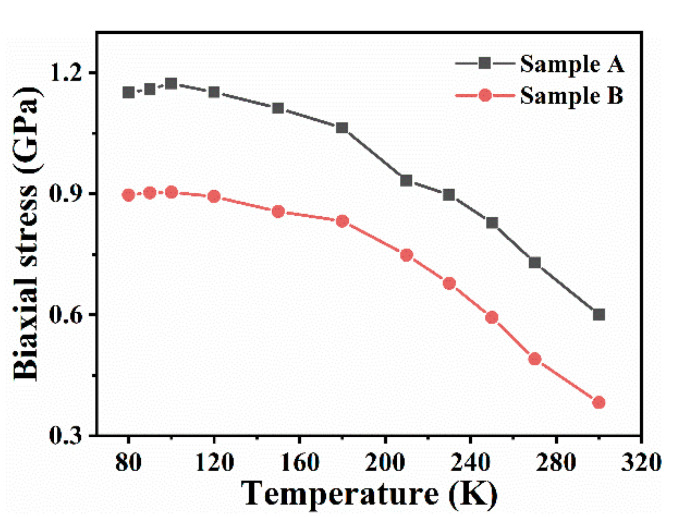
The biaxial stress of AlN/sapphire films as a function of temperature from 80 K to 300 K.

The values of stress σ are calculated from the experimental peak positions ω obtained in this work by using the following Equation [39]:

(1) $$\sigma =\frac{\omega -\omega_{0}}{\kappa }$$

where the stress-shift coefficient *κ* has the value of 4.04 cm^−^^1^/GPa [40] and a stress-free position *ω*_0_ is reported to be 657.4 cm^−^^1^ for E_2_(high) [41,42]. The biaxial stress *σ* values associated with temperature are plotted in Figure 3. As the temperature increased, the value of biaxial stress notably decreased from 1.59 to 0.60 GPa for sample A and slightly reduced from 0.90 to 0.38 GPa for sample B. It can be deduced that nano-patterning leads to very small biaxial stress values. Heating promotes stress release to much lower values for nano-patterned substrate. The data obtained for sample A indicates a compressive stress originating from the interface between the substrate and the epilayer owing to a large lattice mismatch. Imura et al. [43] showed the large thermal expansion coefficient mismatch between AlN and sapphire leads to the tensile stress and cracked epilayer for a layer thickness higher than critical thickness. The disadvantage of excessive stress and cracks can be effectively avoided to much extent by nano-patterning [44]. The substrate patterning is a promising technique to grow ultra-thick AlN films and to improve the structural and optical properties of DUV LEDs.

### 3.3. High-Resolution X-Ray Diffraction Analysis

**Figure 4:**
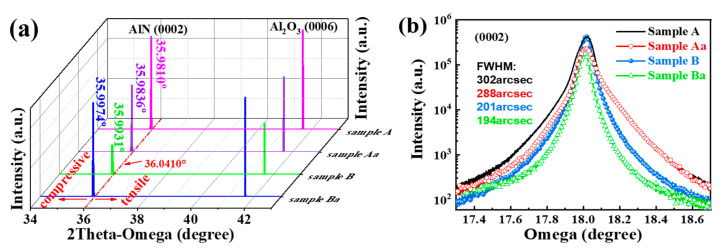
(**a**) The 2θ-ω scan of AlN films by X-ray diffraction (XRD). (**b**) Symmetric (0 0 2) rocking curves of AlN epilayer with different substrates before and after annealing.

The composition and stress related properties of the AlN films grown on various substrates are further analyzed using 2θ-ω X-ray diffraction (XRD) patterns, shown in Figure 4a. The positions of the XRD patterns are calibrated by the Al_2_O_3_ (0006) diffraction plane. The (0002) diffraction planes of samples before (samples A and B) and after (samples Aa and Ba) annealing shift to the 2θ values of 35.9810°, 35.9931°, 35.9836° and 35.9974°, respectively. The variation in 2θ values relative to that of the bulk AlN indicates that the sample is subjected to different stress states. The (0002) diffraction peak shifts to lower (higher) 2θ values with respect to the bulk AlN is used as indication of compressive (tensile) stress in the films [45]. This effect derives from the change in interplanar spacing and diffraction angle that could be clarified by the Bragg diffraction formula. From the above results it can be deduced that all the samples are under compressive stress in agreement with the Raman data. The smaller stress in sample B than in sample A is due to the holes in the nano-patterned substrate, which play the key role in effective stress release.

The values of lattice parameter c and the corresponding residual strain in AlN films grown on various substrates are estimated before and after annealing are given in Table 2. The residual strain as a function of lattice constant in c-axis is calculated using the Equation [46,47]:

(2) $$\varepsilon_{⊥}=\frac{\left(c_{s}-c_{0}\right)}{c_{0}}$$

where *c_s_* and *c*_0_ are the c-axis lattice constants of strained and unstrained AlN films, respectively, and *c*_0_ = 0.4980 nm [46]. The strain values of samples before (samples A and B) and after (samples Aa and Ba) annealing are 0.1606%, 0.1285%, 0.1546% and 0.1164%, respectively. The results demonstrate that the residual strain can be reduced by nano-patterning that further reduces by annealing.

HRXRD is used to test the crystal quality of the AlN films. The symmetric (0002) X-ray rocking curves (XRC) of the AlN epilayer with different substrates before and after annealing are shown in Figure 4b. The (0002) and $\left(10\bar{1}2\right)$ plane of XRC-FWHM values are listed in Table 2. The FWHM of the symmetric (0002) rocking curve for sample A, sample Aa, sample B, and sample Ba are 302, 288, 201, and 194 arcsec, respectively. It reveals that the crystal quality improves after annealing, which promotes grain growth and reduces defects. The higher disorder and defects lead to larger FWHM values, that is further confirmed by the Urbach energy values obtained for both samples (will be discussed in Section 3.5).

### 3.4. Transmission Electron Microscopy Analysis

**Figure 5:**
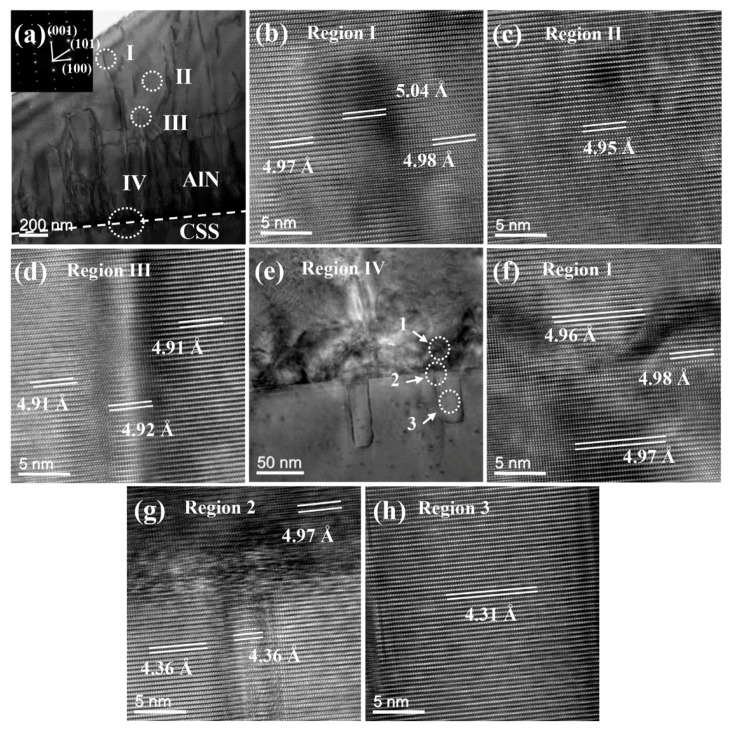
(**a**) A cross-sectional transmission electron microscopy (TEM) image of sample A. High-resolution TEM (HRTEM) images of (**b**) region I; (**c**) region II; (**d**) region III; and (**e**) an interface TEM image in region IV as indicated in (**a**). HRTEM images of (**f**) region 1; (**g**) region 2; and (**h**) region 3 as indicated in (**e**).

TEM is a useful technique for analyzing stress and defects at microscale. The cross-sectional TEM micrographs of the AlN on CSS (sample A) are shown in Figure 5a. To investigate the microstructures in detail, four regions are chosen and marked I, II, III and IV for high-resolution TEM (HRTEM) measurements, as shown in Figure 5b–e, respectively. In region I, the d-spacing at the defect center is up to 5.04 Å, and the d-spacing on both sides of the defect is about 4.98 Å. In the defect-free region II, the d-spacing is 4.95 Å. Although region III also has defects, the d-spacing is significantly smaller than that of regions I and II, that is only 4.91 Å. According to the JCPDS database, the typical d-spacing values of sapphire (0003) and AlN (0001) are 4.33 and 4.98 Å, respectively. The d-spacing is defined as the interatomic spacing or the distance between adjacent planes in the crystalline materials. It can be seen that in the AlN epitaxial layer above the buffer layer, there is a larger d-spacing near the surface of AlN. The Raman and XRD data show that a compressive stress exists in the whole AlN film, but the stress state in the micro-scale region inside the AlN film is different when tested by TEM. The stress is changed from tensile stress in region III to compressive stress in region I. This might be due to the combination and annihilation of the dislocation with the increase of epitaxial thickness during the growth process, which induces the change of stress. Meanwhile, Taniyasu et al. [48] have confirmed that thread dislocations can induce tensile stress in heteroepitaxial AlN layer, which increases with thread dislocation density.

The cross-sectional TEM image of the interface between the AlN buffer layer and CSS are shown in Figure 5e. The various areas in the HRTEM images are marked as region 1, 2 and 3 as shown in Figure 5f–h, respectively. In the AlN buffer layer of region 1 that is affected by the high defect density, the d-spacing varies from 4.96 to 4.98 Å. In region 2, a relative larger lattice mismatch can be seen at the interface, and the d-spacing (4.97 Å) in the AlN buffer layer is extremely close to that of region 1. The d-spacing of U-shaped defect boundaries is 4.36 Å, that matches with the (0003) plane of sapphire. The interior of the U-shaped defect is 4.31 Å, slightly less than the value at the boundary, as shown in region 3 of Figure 5h.

**Figure 6:**
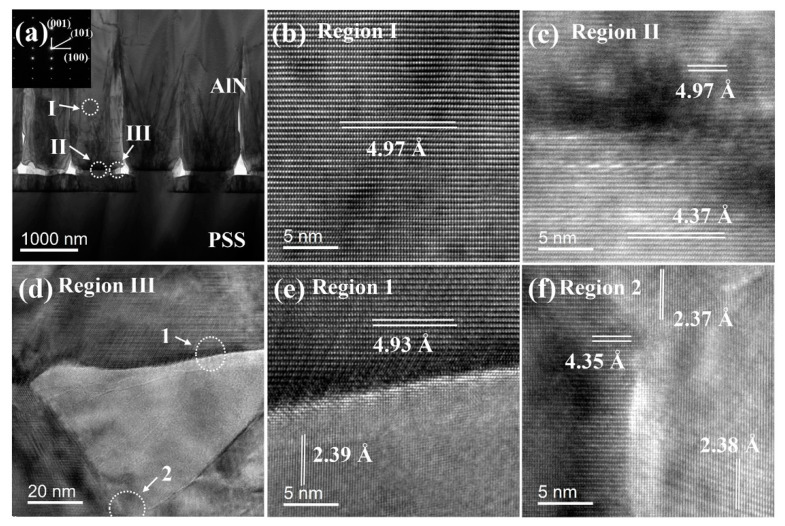
(**a**) A cross-sectional TEM image of sample B. HRTEM images of (**b**) region I; (**c**) region II; and (**d**) region III as indicated in (**a**). HRTEM images of (**e**) region 1; and (**f**) region 2 as indicated in (**d**).

The cross-sectional TEM images of sample B are shown in Figure 6. The interface between the AlN epilayer and the substrate is clearly observed Figure 6a. The AlN buffer layer cannot be observed clearly on the interface, attributed to interdiffusion of atoms during the growth process. Three regions of the AlN epilayer (marked I, II and III) are selected for the HRTEM measurements, as shown in Figure 6b–d, respectively. Here, the d-spacing of region I is 4.97 Å. The same d-spacing value of 4.97 Å for the AlN epilayer is found in region II. The cylindrical unit of NPSS has 4.37 Å d-spacing, corresponding to the (0003) plane of sapphire. In order to observe the d-spacing more clearly, region III is subdivided into regions 1 and 2, as shown in Figure 6e,f. In region 1, the d-spacing of AlN is 4.93 Å, indicating that the initial growth stage is subjected to a large tensile stress. Subsequently, the tensile stress is released due to the key-shaped holes as the growth progresses and finally the stress changes to compressive. Meanwhile, in regions 1 and 2, it can be observed that there exists a 2.37~2.39 Å d-spacing in the vertical direction, which is the $\left(10\bar{1}1\right)$ plane of AlN. The d-spacing of 4.35 Å in region 2 is consistent with 4.37 Å in region II, both of which belong to the (0003) plane of sapphire. The variation of internal stress in the NPSS sample is small compared with the CSS sample. The key-shaped holes in NPSS sample make the internal stress release more easily.

### 3.5. Optical Transmission Spectroscopy Analysis

**Figure 7:**
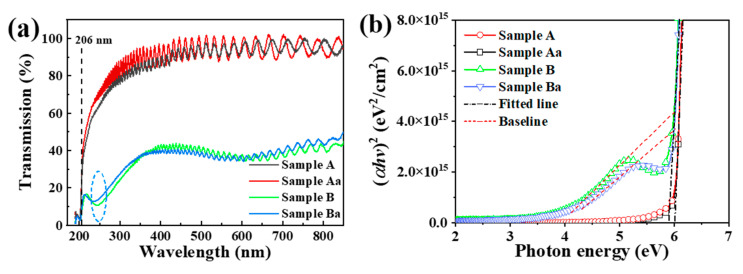
(**a**) Transmission spectra of AlN/sapphire before and after annealing. (**b**) Plots of (αhυ)^2^ against photon energy (hυ) for AlN films.

As the crystal quality and stress are found to be corelated, calculating Urbach energy values can give further insight into crystal quality. Urbach energy can be calculated from UV-Vis absorption/transmission spectroscopy. The stress and optical properties can be corelated in this way. Annealing is also an important factor that can lead to defect healing and lowering the stress values in AlN. To calculate the Urbach energy values and investigate the effect of substrate nano-patterning and annealing on the optical transmission of the AlN epilayer, we used a dual-beam UV-Vis spectrophotometer to measure transmission spectra with the vertical incidence in the wavelength of 190–850 nm. Figure 7a shows that the transmission spectra obtained by two types of sapphire substrates are greatly different. The transmittance for CSS is higher than that of the NPSS sample, especially in the region 220–245 nm where a local minimum is observed at 235 nm for nano-patterned substrate. That may be mainly caused by the increased light scattering and trapping in the nano-holes of NPSS near the interface. The material is nearly transparent above the 210 nm wavelength region. However, the transmittance is extremely weak and drops dramatically when below 210 nm, indicating that the absorption edge exists there. Both samples exhibit very sharp wavelength cutoff curves at ~206 nm, but only the as-grown AlN exhibits a local minimum at the cutoff corner, indicating that the crystal quality of the annealed sample is better than that of the as-grown sample [49].

For semiconductor materials with direct bandgap, the relationship between the absorption coefficient and the photon energy is determined by the Tauc empirical relation [50,51]:

(3) $$\alpha h\upsilon =B{\left(h\upsilon -E_{g}\right)}^{1/2}$$

where the absorption coefficient *α* = 2.303 A/d (A is absorbance, and d denotes the thickness of the semiconductor material), *B* indicates a constant, *hυ* is photon energy, and *E_g_* is the bandgap of the semiconductor. The linear absorption edge is obtained by plotting (*αhυ*)^2^ versus *hυ*, as shown in Figure 7b. At the intersection of the linear absorption edge and *hv*, that is (*αhυ*)^2^ = 0, the corresponding *E_g_* can be acquired to be 6.03 eV. Due to a considerable absorbance at energies below *E_g_*, the bandgap energies for sample B and Ba are measured by the baseline approach derived by Makula et al. [52]. An intersection of the two fitting lines enables the bandgap energy to be obtained directly from the plot, and the *E_g_* of sample B and Ba are estimated as 6.01 eV and 6.00 eV, respectively. The difference of bandgap values is caused by the compressive stress in the samples. After annealing, the compressive stress in the sample is partially eliminated, which makes the atomic spacing in the crystal increase the bandgap to redshift.

**Figure 8:**
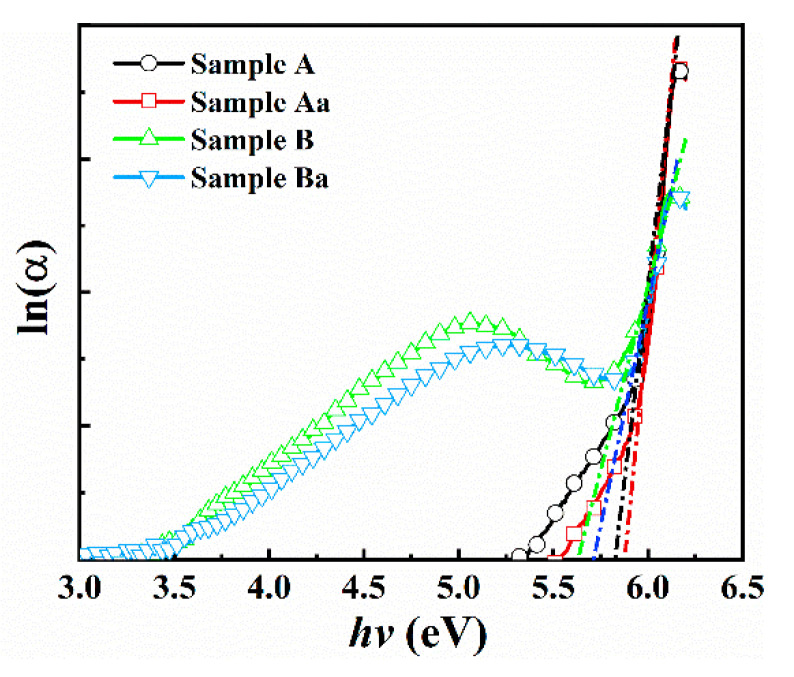
The relation between ln(*α*) and photon energy (*hυ*) for samples.

Regarding the energy range below the bandgap the absorption coefficient shows the nonzero values that are characterized by the Urbach tail [53]. Urbach energies (*E_U_*) of the samples can be calculated by plotting the absorption coefficient (*α*) as a function of photon energy (*hυ*). For semiconductors, the Urbach tail is related to the degree of crystal disorder and defects [54]. Urbach energies can be extracted according to the following Equation [55]:

(4) $$\alpha =\alpha_{0}exp\left(\frac{h\upsilon }{E_{U}}\right)$$

where *α*_0_ is a constant and *E_U_* is the width of the localized band-tail states. Generally, samples with low levels of impurities, defects and electron-phonon interactions tend to have a small *E_U_*. Figure 8 shows that the *E_U_* of the sample A, sample Aa, sample B and sample Ba are 0.27, 0.25, 0.23 and 0.18 eV, respectively. The decreasing trend in Eu values after annealing is due to improvement in crystal quality and increased long range order [56]. The lower Urbach energy indicate lower defect density in AlN on nano-patterned substrate, in agreement with Raman data. Nano-patterning decreases defect density and allows stress relaxation in the nano-hole. Meanwhile, annealing helps reduce the stress by defect healing by atomic rearrangement.

### 3.6. Temperature-Dependent Spectroscopic Ellipsometry Analysis

**Figure 9:**
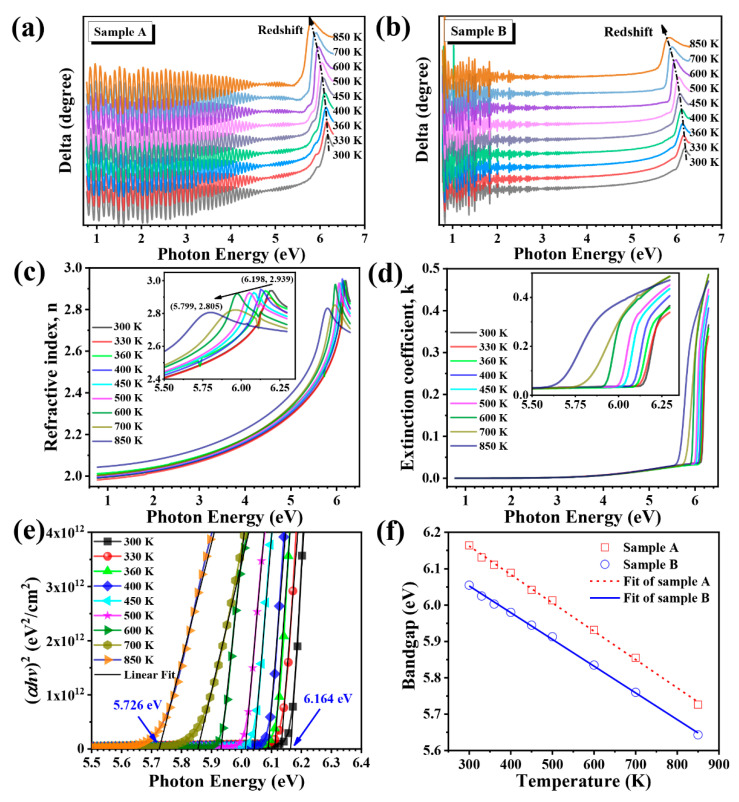
The experimental data of the delta (Δ) graph with the incident angle of 70° in the temperature range of 300 K to 850 K: (**a**) sample A and (**b**) sample B. Fitted optical constants (**c**) refractive index and (**d**) extinction coefficients of sample A with temperature varied from 300 K to 850 K. (**e**) The square of the absorption coefficient (*αhυ*)^2^ vs. photon energy (*hυ*) for sample A at 300–850 K. (**f**) The variation of bandgap (*E_g_*) vs. temperature (T) of sample A fitted by the Varshni empirical equation in the range 300–850 K.

To take a deeper insight into temperature induced variations in optical properties, two samples are investigated by VASE. The parameters of spectroscopic ellipsometry include the amplitude ratio Psi (ψ) and phase difference Delta (Δ), where Δ is known as a function of photon energy. Figure 9a,b shows the experimental results of Δ obtained by ellipsometry data with the incident angle of 70° in the temperature range of 300–850 K. It can be clearly noticed that with the increase of temperature, the value of Δ decreases; that should lead to bandgap reduction. Figure 9a,b shows that the interference oscillation of sample A is relatively regular, while it is irregular for sample B. There is a strong light scattering and trapping effect at the interface between the nano-patterned substrate and epitaxial layer, which also supports the conclusions from the transmission spectra.

The thickness and surface roughness values of sample A are obtained by fitting SE data that are ~3.02 μm and 2.50 nm, respectively. The AlN buffer layer is ~16 nm. The refractive index (n) and extinction coefficient (k) as a function of photon energy from 0.775 to 6.295 eV can be obtained from the ψ and Δ data in the temperature range of 300–850 K as shown in Figure 9c,d. The n and k values vary with increasing temperature as shown clearly in the insets. As the temperature increases, the peak of n redshifts from (6.198, 2.939) to (5.799, 2.805) in the high-energy region, and the n value is higher at high temperatures in the low-energy regions. The extinction coefficients move to the low-energy direction as the temperature increases. An absorption tail is observed in the high-energy region for extinction coefficients, attributed to the existence of defects.

In order to calculate the direct bandgap of AlN, the formula of the absorption coefficient α can be derived from the extinction coefficient *k* and wavelength *λ*, that is α=4πk/λ [57]. Combining with the Equation (3), the bandgap *E_g_* of AlN films can be estimated by linear extrapolation of a plot between (*αhυ*)^2^ versus *hυ* as described in Figure 9e. The values of bandgap change from 6.16 to 5.73 eV for sample A and from 6.06 to 5.64 eV for sample B in the temperature range of 300–850 K. This behavior can be explained as the increase in interatomic spacing because of increasing thermal energy. As the interatomic spacing increases, the potential of the electrons decreases leading to reduction in the bandgap [58]. The bandgap reduction with increasing temperature could also be explained by thermal expansion and electron–phonon interactions [59,60].

The relationship of temperature (*T*) and the bandgap *E_g_* has been first proposed by Varshni and expressed as [61]:

(5) $$E_{g}=E_{0}-\frac{aT^{2}}{T+b}$$

where *E*_0_ is the transition energy at 0 K, while *a* and *b* are the Varshni coefficients. The hollow points represent experimental data and the dash (solid) lines represent the least-square fit to the experimental data using Varshni empirical equation as shown in Figure 9f. The parameters obtained from the best fit are *E*_0_ = 6.24 eV, *a* = 8.73 × 10^−^^4^ eV/K and *b* = 857 K for sample A, and *E*_0_ = 6.19 eV, *a* = 7.84 × 10^−^^4^ eV/K and *b* = 672 K for sample B. The bandgap variation reflects the redshift with the increase in temperature, which is quite typical in nitride semiconductors.

## 4. Conclusions

The influence of temperature on stress and optical properties of the AlN epilayers on flat and nanopatterned substrates has been studied by various characterization techniques. Both samples show compressive stress, that decreases with temperature as estimated by the *E*_2_(high) Raman peak of AlN. The value of biaxial stress decreased from 1.59 to 0.60 GPa for sample A and from 0.90 to 0.38 GPa for sample B in the temperature range 80–300 K. It is found that nano-patterning leads to lower disorder and defects in AlN epilayer due to the effective stress release of the epilayer in nano-holes, which is confirmed by XRD measurement. Furthermore, annealing is found to reduce the residual strain by defect healing. The change of stress state in the micro-scale region and interface is characterized by TEM. The stress changes from tensile at the interface to compressive on the surface. The key-shaped holes characteristic of NPSS growth are more conducive to the release of internal stress. The calculated Urbach energies show that the lattice disorder and defects can be reduced by annealing; as well, the sample of NPSS has better crystal quality. The refractive index and extinction coefficient as a function of photon energy from 0.775 to 6.295 eV were obtained by SE. The values of bandgap for samples A and B change from 6.16 to 5.73 eV and from 6.06 to 5.64 eV in the temperature range of 300–850 K, respectively. The analysis method of temperature-dependent stress, optical constant and optical bandgap has been proposed, which enables us to predict the thermo-optic effect and optimize the optical properties of AlN-based high power devices for temperatures up to 850 K.

## Figures and Tables

**Table 1:** Summary of growth information and thickness of AlN samples.

Sample No.	Growth Mode	Precursor	Layer	Thickness (µm)	RSM (nm)
Sample A	Continue	TMAl, NH_3_	AlN/AlN/CSS	3.02/0.015/435	2.32
Sample B	Continue + Pulse	TMAl, NH_3_	AlN/AlN/NPSS	5.12/0.015/435	2.36

**Table 2:** The structural parameters and dislocation density values obtained by XRD of the samples.

Sample No.	Lattice Constant, c (Å)	Strain, ε⊥ (%)	FWHM (Arcsec)	
			(0002)	$\left(10\bar{1}2\right)$
Sample A	4.9880	0.1606	302	464
Sample Aa	4.9877	0.1546	288	385
Sample B	4.9864	0.1285	201	410
Sample Ba	4.9858	0.1164	194	320
Unstrained AlN	4.9800	0	-	-

## Data Availability

The data presented in this study are available on request from the corresponding author.

## References

[1] Yamashita H., Fukui K., Misawa S., Yoshida S. (1979). Optical properties of AlN epitaxial thin films in the vacuum ultraviolet region. J. Appl. Phys..

[2] Slack G.A., Tanzilli R.A., Pohl R.O., Vandersande J.W. (1987). The intrinsic thermal conductivity of AIN. J. Phys. Chem. Solids.

[3] Kim T., Kim J., Dalmau R., Schlesser R., Preble E., Jiang X. (2015). High-temperature electromechanical characterization of AlN single crystals. IEEE Trans. Ultrason. Ferroelectr. Freq. Control..

[4] Al Tahtamouni T.M., Lin J.Y., Jiang H.X. (2012). High quality AlN grown on double layer AlN buffers on SiC substrate for deep ultraviolet photodetectors. Appl. Phys. Lett..

[5] Sotnikov A., Schmidt H., Weihnacht M., Smirnova E., Chemekova T., Makarov Y. (2010). Elastic and piezoelectric properties of AlN and LiAlO_2_ single crystals. IEEE Trans. Ultrason. Ferroelectr. Freq. Control..

[6] Xu Y.N., Ching W.Y. (1993). Electronic, optical, and structural properties of some wurtzite crystals. Phys. Rev. B Condens. Matter.

[7] Levinshtein M.E., Rumyantsev S.L., Shur M.S. (2001). Properties of advanced semiconductor materials: GaN, AIN, InN, BN, SiC, SiGe. Scitech Book News.

[8] Yim W.M., Paff R.J. (1974). Thermal expansion of AlN, sapphire, and silicon. J. Appl. Phys..

[9] Sun W.H., Zhang J.P., Yang J.W., Maruska H.P., Khan M.A., Liu R., Ponce F.A. (2005). Fine structure of AlN/AlGaN superlattice grown by pulsed atomic-layer epitaxy for dislocation filtering. Appl. Phys. Lett..

[10] Sun W.H., Zhang J.P., Adivarahan V., Chitnis A., Shatalov M., Wu S., Mandavilli V., Yang J.W., Khan M.A. (2004). AlGaN-based 280 nm light-emitting diodes with continuous wave powers in excess of 1.5 mW. Appl. Phys. Lett..

[11] Katagiri Y., Kishino S., Okuura K., Miyake H., Hiramatu K. (2009). Low-pressure HVPE growth of crack-free thick AlN on a trench-patterned AlN template. J. Cryst. Growth.

[12] Jain R., Sun W., Yang J., Shatalov M., Hu X., Sattu A., Lunev A., Deng J., Shturm I., Bilenko Y. (2008). Migration enhanced lateral epitaxial overgrowth of AlN and AlGaN for high reliability deep ultraviolet light emitting diodes. Appl. Phys. Lett..

[13] Kozlovskiy A., Kenzhina I., Alyamova Z.A., Zdorovets M. (2019). Optical and structural properties of AlN ceramics irradiated with heavy ions. Opt. Mater..

[14] Goto T., Tsuneyoshi J., Kaya K., Hirai T. (1992). Preferred orientation of AlN plates prepared by chemical vapour deposition of AlCl_3_ + NH_3_ system. J. Mater. Sci..

[15] Choi J.S., Ko D.W., Cho S.M., Lee S.T., Chang J.H. (2020). Residual strain in the AlN layers deposited by reactive-gas pulsed sputtering deposition. Trans. Electr. Electron. Mater..

[16] Kolaklieva L., Chitanov V., Szekeres A., Antonova K., Terziyska P., Fogarassy Z., Petrik P., Mihailescu I., Liviu D. (2019). Pulsed laser deposition of aluminum nitride films: Correlation between mechanical, optical, and structural properties. Coatings.

[17] Rong X., Wang X., Chen G., Pan J., Wang P., Liu H., Xu F., Tan P., Shen B. (2016). Residual stress in AlN films grown on sapphire substrates by molecular beam epitaxy. Superlattices Microstruct..

[18] Kumada T., Ohtsuka M., Fukuyama H. (2015). Influence of substrate temperature on the crystalline quality of AlN layers deposited by RF reactive magnetron sputtering. Aip Adv..

[19] Freitas J.A., Culbertson J.C., Mastro M.A., Kumagai Y., Koukitu A. (2012). Structural and optical properties of thick freestanding AlN films prepared by hydride vapor phase epitaxy. J. Cryst. Growth.

[20] Paduano Q., Weyburne D. (2003). Two-step process for the metalorganic chemical vapor deposition growth of high quality AlN films on sapphire. Jpn. J. Appl. Phys..

[21] Bryant W.A. (1977). The fundamentals of chemical vapour deposition. J. Mater. Sci..

[22] Yang Z., Hao J. (2016). Progress in pulsed laser deposited two-dimensional layered materials for device applications. J. Mater. Chem. C.

[23] Ambacher O. (1998). Growth and applications of Group III-nitrides. J. Phys. D Appl. Phys..

[24] Kovalenkov O., Soukhoveev V., Ivantsov V., Usikov A., Dmitriev V. (2005). Thick AlN layers grown by HVPE. J. Cryst. Growth.

[25] Ambacher O., Brandt M.S., Dimitrov R., Metzger T., Stutzmann M., Fischer R.A., Miehr A., Bergmaier A., Dollinger G. (1996). Thermal stability and desorption of Group III nitrides prepared by metal organic chemical vapor deposition. J. Vac. Sci. Technol. B.

[26] McMurdie H.F., Morris M.C., Evans E.H., Paretzkin B., Wong-Ng W., Ettlinger L., Hubbard C.R. (1986). Standard X-ray diffraction powder patterns from the JCPDS research associateship. Powder Diffr..

[27] Long H., Dai J., Zhang Y., Wang S., Tan B., Zhang S., Xu L., Shan M., Feng Z.C., Kuo H.-C. (2019). High quality 10.6 μm AlN grown on pyramidal patterned sapphire substrate by MOCVD. Appl. Phys. Lett..

[28] Wu Z., Yan J., Guo Y., Zhang L., Lu Y., Wei X., Wang J., Li J. (2019). Study of the morphology evolution of AlN grown on nano-patterned sapphire substrate. J. Semicond..

[29] Zhang L., Xu F., Wang J., He C., Guo W., Wang M., Sheng B., Lu L., Qin Z., Wang X. (2016). High-quality AlN epitaxy on nano-patterned sapphire substrates prepared by nano-imprint lithography. Sci. Rep..

[30] Zubar T.I., Fedosyuk V.M., Trukhanov S.V., Tishkevich D.I., Michels D., Lyakhov D., Trukhanov A.V. (2020). Method of surface energy investigation by lateral AFM: Application to control growth mechanism of nanostructured NiFe films. Sci. Rep..

[31] Tishkevich D., Grabchikov S., Zubar T., Vasin D., Trukhanov S., Vorobjova A., Yakimchuk D., Kozlovskiy A., Zdorovets M., Giniyatova S. (2020). Early-Stage Growth Mechanism and Synthesis Conditions-Dependent Morphology of Nanocrystalline Bi Films Electrodeposited from Perchlorate Electrolyte. Nanomaterials.

[32] Deng Y., Kong Y., Zheng Y., Zhou C., Xi D., Chen P., Gu S., Shen B., Zhang R., Han P. (2005). Study on strain and piezoelectric polarization of AlN thin films grown on Si. J. Vac. Sci. Technol. A.

[33] Harima H. (2002). Properties of GaN and related compounds studied by means of Raman scattering. J. Phys. Condens. Matter.

[34] Severino A., Iucolano F. (2016). Impact of growth conditions on stress and quality of aluminum nitride (AlN) thin buffer layers. Phys. Status Solidi B.

[35] Sumathi R. (2013). Bulk AlN single crystal growth on foreign substrate and preparation of free-standing native seeds. CrystEngComm.

[36] Kuball M., Hayes J.M., Shi Y., Edgar J.H., Prins A.D., van Uden N.W.A., Dunstan D.J. (2001). Raman scattering studies on single-crystalline bulk AlN: Temperature and pressure dependence of the AlN phonon modes. J. Cryst. Growth.

[37] Menéndez J., Cardona M. (1984). Temperature dependence of the first-order Raman scattering by phonons in Si, Ge, and a-Sn: Anharmonic effects. Phys. Rev. B.

[38] Xie N., Xu F., Wang J., Sun Y., Liu B., Zhang N., Lang J., Fang X., Ge W., Qin Z. (2020). Stress evolution in AlN growth on nano-patterned sapphire substrates. Appl. Phys. Express.

[39] Wang M.X., Xu F.J., Xie N., Sun Y.H., Liu B.Y., Ge W.K., Kang X.N., Qin Z.X., Yang X.L., Wang X.Q. (2019). High-temperature annealing induced evolution of strain in AlN epitaxial films grown on sapphire substrates. Appl. Phys. Lett..

[40] Yang S., Miyagawa R., Miyake H., Hiramatsu K., Harima H. (2011). Raman scattering spectroscopy of residual stresses in epitaxial AlN films. Appl. Phys. Express.

[41] Prokofyeva T., Seon M., Vanbuskirk J., Holtz M., Nikishin S.A., Faleev N.N., Temkin H., Zollner S. (2001). Vibrational properties of AlN grown on (111)-oriented silicon. Phys. Rev. B.

[42] Davydov V.Y., Kitaev Y.E., Goncharuk I.N., Smirnov A.N., Graul J., Semchinova O., Uffmann D., Smirnov M.B., Mirgorodsky A.P., Evarestov R.A. (1998). Phonon dispersion and Raman scattering in hexagonal GaN and AlN. Phys. Rev. B.

[43] Imura M., Nakano K., Fujimoto N., Okada N., Balakrishnan K., Iwaya M., Kamiyama S., Amano H., Akasaki I., Noro T. (2007). Dislocations in AlN epilayers grown on sapphire substrate by high-temperature metal organic vapor phase epitaxy. Jpn. J. Appl. Phys..

[44] Wang T.Y., Tasi C.T., Lin K.Y., Ou S.L., Horng R.H., Wuu D.S. (2018). Surface evolution and effect of V/III ratio modulation on etch-pit-density improvement of thin AlN templates on nano-patterned sapphire substrates by metalorganic chemical vapor deposition. Appl. Surf. Sci..

[45] Saron K.M.A., Hashim M.R., Farrukh M.A. (2012). NH_3_-free growth of GaN nanostructure on n-Si (111) substrate using a conventional thermal evaporation technique. J. Cryst. Growth.

[46] Chen X.W., Jia C.H., Chen Y.H., Wang H.T., Zhang W.F. (2014). Epitaxial growth and optical properties of Al- and N-polar AlN films by laser molecular beam epitaxy. J. Phys. D Appl. Phys..

[47] Park B.G., Saravana Kumar R., Moon M.L., Kim M.D., Kang T.W., Yang W.C., Kim S.G. (2015). Comparison of stress states in GaN films grown on different substrates: Langasite, sapphire and silicon. J. Cryst. Growth.

[48] Taniyasu Y., Kasu M., Makimoto T. (2007). Threading dislocations in heteroepitaxial AlN layer grown by MOVPE on SiC (0001) substrate. J. Cryst. Growth.

[49] Sun W.H., Yang J.W., Zhang J.P., Gaevski M.E., Chen C.Q., Li J.W., Gong Z., Su M., Asif Khan M. (2005). n-Al_0.75_Ga_0.25_N epilayers for 250 nm emission ultraviolet light emitting diodes. Phys. Status Solidi C.

[50] Ou S.L., Wuu D.S., Fu Y.C., Liu S.P., Horng R.H., Liu L., Feng Z.C. (2012). Growth and etching characteristics of gallium oxide thin films by pulsed laser deposition. Mater. Chem. Phys..

[51] Khedmi N., Ben Rabeh M., Kanzari M. (2014). Structural morphological and optical properties of SnSb_2_S_4_ thin films grown by vacuum evaporation method. J. Mater. Sci. Technol..

[52] Makula P., Pacia M., Macyk W. (2018). How To Correctly Determine the Band Gap Energy of Modified Semiconductor Photocatalysts Based on UV-Vis Spectra. J. Phys. Chem. Lett..

[53] Urbach F. (1953). The long-wavelength edge of photographic sensitivity and of the electronic absorption of solids. Phys. Rev..

[54] Wu Y., Wei C., Li X., Li Y., Qiu S., Shen W., Cai B., Sun Z., Yang D., Deng Z. (2018). In situ passivation of PbBr_6_^4–^ octahedra toward blue luminescent CsPbBr_3_ nanoplatelets with near 100% absolute quantum yield. ACS Energy Lett..

[55] Studenyak I., Kranjčec M., Kurik M. (2014). Urbach rule in solid state physics. Int. J. Opt. Appl..

[56] Saleem M.F., Haleem Y.A., Sun W., Ma L., Wang D. (2020). Surface-enhanced resonance Raman scattering in partially oxidized thin copper film. J. Raman Spectrosc..

[57] Fujiwara H. (2007). Introduction to Spectroscopic Ellipsometry.

[58] Wei W., Wang J., Liu Y., Peng Y., Maraj M., Peng B., Wang Y., Sun W. (2020). Effects of thermal annealing on optical properties of Be-Implanted GaN thin films by spectroscopic ellipsometry. Crystals.

[59] Guo Q., Yoshida A. (1994). Temperature dependence of band gap change in InN and AlN. Jpn. J. Appl. Phys..

[60] Nam K.B., Li J., Lin J.Y., Jiang H.X. (2004). Optical properties of AlN and GaN in elevated temperatures. Appl. Phys. Lett..

[61] Varshni Y.P. (1967). Temperature dependence of the energy gap in semiconductors. Physica.

